# Automated temporal analysis reveals stress-induced protracted feeding regulated by mesolimbic dopamine in mice

**DOI:** 10.3389/fnins.2026.1959447

**Published:** 2026-10-20

**Authors:** Xiaokai Guo, Yusuke Fujioka, Kaori Kawai, Shinsuke Ishigaki

**Affiliations:** 1 Molecular Neuroscience Research Center, Shiga University of Medical Science, Ōtsu, Shiga, Japan; 2 Hebei Chest Hospital, Shijiazhuang, Hebei, China; 3 Department of Aging Neurobiology, Center for Development of Advanced Medicine for Dementia, National Center for Geriatrics and Gerontology, Ōbu, Aichi, Japan

**Keywords:** AI-based system, automated behavioral analysis, dopamine, feeding behavior patterns, fixated feeding, protracted feeding, psychosocial stress, reward system

## Abstract

Feeding behavior is altered in neuropsychiatric disorders, and environmental stressors also shape it. In our previous study, mice exposed to multiple stressors developed feeding behavior patterns characterized by a deviated bait approach, which we defined as “fixated feeding,” together with impairment of the mesolimbic dopamine system. Here we examined the temporal organization of feeding behavior using an AI-based analytical framework built on DeepLabCut and SimBA. Mice subjected to social isolation or intermittent high-fat diet (HFD) exposure showed prolonged feeding bouts, a pattern we define as “protracted feeding.” Dopamine administration into the nucleus accumbens (NAcc) shell attenuated diet- and isolation-induced protracted feeding. Physical restraint stress alone did not induce protracted feeding, but mosapride treatment prolonged feeding bouts in restrained mice. Restraint-associated peripheral autonomic and gastrointestinal states may therefore influence the expression of stress-related temporal feeding deviations. In the VTA-DREADD cohort, the same temporal analysis showed that inhibition of VTA dopaminergic activity prolonged feeding bouts. This extends the fixated-feeding circuit framework from the spatial to the temporal domain. These deviations were not consistently accompanied by changes in the amount consumed or in metabolic factors, so alterations in temporal feeding patterns may reflect perturbation of a stress-associated pathway in the mesolimbic dopamine system. Protracted feeding, together with fixated feeding, may serve as a candidate behavioral readout of stress-associated reward-system dysfunction.

## 1 Introduction

Psychosocial stress is a major determinant of behavioral alterations, including impairments in eating patterns (Adam and Epel, 2007). Observations that certain individuals habitually choose the same foods or struggle to resist specific snacks prompted us to investigate the mechanisms that regulate feeding behavior, irrespective of the quantity consumed. Stress is linked to both overeating and anorexia, so the amount consumed is an unreliable index of how stress acts on the feeding system. Feeding behavior is determined by how much food is consumed and also by qualitative features such as food choice and feeding patterns, including when and how feeding occurs (Sasaki, 2017). These qualitative aspects arise from the interaction of homeostatic hypothalamic mechanisms with hedonic and motivational circuits, including the mesolimbic dopamine pathways (Lutter and Nestler, 2009; Berridge et al., 2010; Salamone and Correa, 2012). In our previous study, psychosocial stressors consistently induced hyporesponsiveness in the mesolimbic dopamine system, which altered the qualitative features of feeding and produced fixated feeding preferences (Fujioka et al., 2024). Deviations in feeding behavior patterns may therefore serve as sensitive readouts for stress conditions and neuropsychiatric disorders. Our previous work used a real-time monitoring system to identify spatial fixated feeding, but the temporal microstructure of these behaviors remained unclear.

Behavioral assessment is central to neurobehavioral studies and is used to evaluate phenotypes from cognitive ability to neurological disorders (Takumi et al., 2020; Pentkowski et al., 2021; Kishi et al., 2025). Much of this work still depends on manual scoring, which is subject to observer fatigue and bias and does not scale well. Automated frameworks address these limits: they increase experimental throughput and reduce the burden on both investigators and animals. Markerless pose estimation and supervised behavioral classification, including DeepLabCut and SimBA, now allow complex behavioral states to be quantified reproducibly, frame by frame (Mathis et al., 2018; Goodwin et al., 2024). Such approaches belong to computational neuroethology, in which pose estimation and machine-learning classifiers are used to extract reproducible behavioral structure from video (Pereira et al., 2020).

In the present study, we quantified the temporal dynamics of feeding under the same stress paradigms used in our previous work, including social isolation, intermittent HFD exposure, and physical restraint, using AI-based pose estimation and behavioral classification. We chose these paradigms because they produced divergent quantitative feeding outcomes in our previous study, whereas spatial feeding-pattern abnormalities and impaired NAcc shell dopamine responses were shared across the stress conditions. We kept the conceptual framework of fixated feeding but extended it from spatial bias to temporal organization. Our question was whether protracted feeding provides a sensitive behavioral readout of stress-impaired reward-system function.

## 2 Methods

### 2.1 Mice and stress models

C57BL/6J and DAT-Cre mice were used in this study. The intermittent HFD and social isolation paradigms are illustrated in Figures 1A, B, and the AI-based analysis workflow and its validation against manual scoring are shown in Figures 1C, D. Results for the intermittent HFD and social isolation cohorts are shown in Figure 2, those for the physical restraint and mosapride cohorts in Figure 3, and those for the intra-NAcc shell dopamine and DREADD cohorts in Figure 4. The stress paradigms were based on those used in our previous study, including social isolation, intermittent high-fat diet (HFD), and physical restraint stress (Fujioka et al., 2024). Whereas the previous study primarily assessed spatial fixated-feeding patterns and reward-pathway dysfunction, the present study focused on temporal feeding-bout duration and protracted feeding. For the social isolation model, wild-type C57BL/6J mice were randomly assigned to either a social isolation group or a group-housed control group. Control mice were housed in groups of four under standard animal care conditions, whereas mice in the social isolation group were housed individually for 7 days before behavioral recording. For the intermittent HFD model, 6-week-old C57BL/6J mice were randomly assigned to either an intermittent HFD group or a control group. Throughout the housing and stress-exposure periods, mice in all groups, including all control groups, had ad libitum access to standard chow and to water. Standard chow remained available during the 2-h HFD access windows, and food and water were unavailable only during the 2-h daily immobilization periods in the restraint group. The food deprivation applied immediately before each behavioral recording is described in section “2.2 Behavioral video recordings and AI-based temporal analysis.” The intermittent HFD group was given access to HFD for 2 h every other day for 2 weeks, whereas control mice were maintained on standard chow. For the physical restraint stress model, 7-week-old C57BL/6J mice were randomly assigned to either a restraint stress group or an unrestrained control group. Mice in the restraint group were completely immobilized using disposable mouse DecapiCone restrainers for 2 h per day for five consecutive days before behavioral recording, as described previously (Fujioka et al., 2024). DAT-Cre mice were used for chemogenetic inhibition of VTA dopaminergic neurons. The DAT-Cre line used was B6.SJL-Slc6a3tm1.1(cre)Bkmn/J (The Jackson Laboratory), which carries Cre recombinase inserted into the 3′ untranslated region of the endogenous dopamine transporter (Slc6a3) locus. This design leaves both Slc6a3 alleles functional and restricts Cre expression to dopamine-transporter-expressing neurons, which makes the line suitable for targeting VTA dopaminergic neurons (Bäckman et al., 2006). For the DAT-Cre DREADD cohort, AAV-hSyn-FLEX-hM4Di/mCherry was injected into the VTA of 6-week-old DAT-Cre mice, and CNO or vehicle was administered before behavioral recording. Behavioral recordings from this cohort were re-analyzed from previously generated datasets to assess temporal feeding-bout duration in the present study. Male and female mice were included as indicated in the individual figure legends. The intermittent HFD, social isolation and physical restraint cohorts comprised male and female mice in balanced or nearly balanced numbers, whereas the mosapride, intra-NAcc shell dopamine and DREADD cohorts comprised male mice only; the reasons are given in sections “2.5 Stereotaxic surgery and pharmacological manipulation” and “2.6 Mosapride administration” and in the corresponding figure legends. Per-group sample sizes, sex composition, statistical tests, and exact p values are reported in the corresponding figure legends. The physical restraint stress and mosapride cohort (Figures 3D–F) was newly acquired for the present study. The intermittent HFD and social isolation cohorts (Figure 2), the physical restraint only cohort (Figures 3A–C), and the intra-NAcc shell dopamine and DREADD cohorts (Figure 4) consisted of behavioral recordings originally obtained in our previous study (Fujioka et al., 2024). These recordings were re-analyzed here exclusively for temporal feeding-bout organization, an outcome not examined in that study. Total food intake during the same 4-h recording sessions was reported for these cohorts in that study (Fujioka et al., 2024; Figures 3A, B for the intermittent HFD, social isolation and physical restraint cohorts; Supplementary Figures 3A, B for the intra-NAcc shell dopamine cohorts) and was therefore not re-measured here; intake was measured de novo only in the newly acquired mosapride cohort (Figure 3F). There is therefore no overlap in the analyzed outcomes between the two studies. Within each experiment, animals were randomly assigned to groups using a computer-generated randomization sequence. Sample sizes were based on those used in our previous study (Fujioka et al., 2024).

**FIGURE 1:**
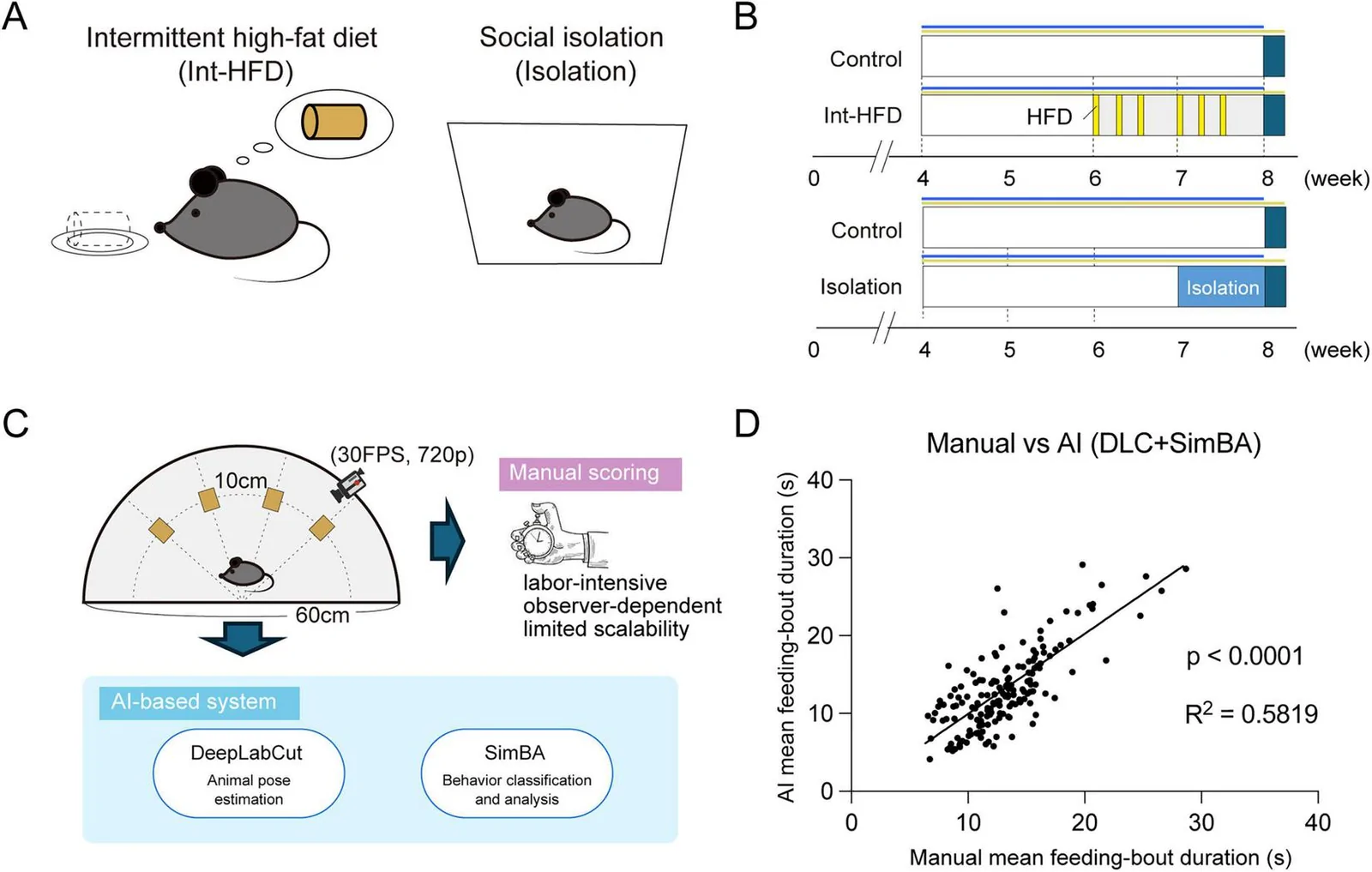
AI-based temporal analysis extends the assessment of feeding behavior patterns from spatial fixation to temporal structure. **(A,B)** Study paradigms for intermittent HFD and social isolation are shown. Mice in the intermittent HFD group accessed an HFD for 2 h every other day for 2 weeks, whereas mice in the social isolation group were maintained alone for 1 week. In the timelines, blue and yellow lines indicate water and standard chow availability and the dark box the 4-h recording session, which followed 4 h of food deprivation with water available (not drawn to scale); during the session chow was available in the arena bait containers and water was not. **(C)** Experimental scheme for AI-based feeding analysis. Four bait containers containing standard chow diet were affixed in the arena, and mouse behavior was recorded at 30 frames per second. DeepLabCut was used for markerless pose estimation, and SimBA was used for automated behavioral classification. **(D)** Relationship between AI-based analysis and manual scoring. Each point represents one mouse and shows the manually determined mean feeding-bout duration and the corresponding AI-derived mean feeding-bout duration. Data were pooled across the intermittent HFD, social isolation, and physical restraint experiments; the animal used for classifier training was excluded. Linear regression analysis yielded R^2^ = 0.5819 (*p* < 0.0001, *n* = 170).

**FIGURE 2:**
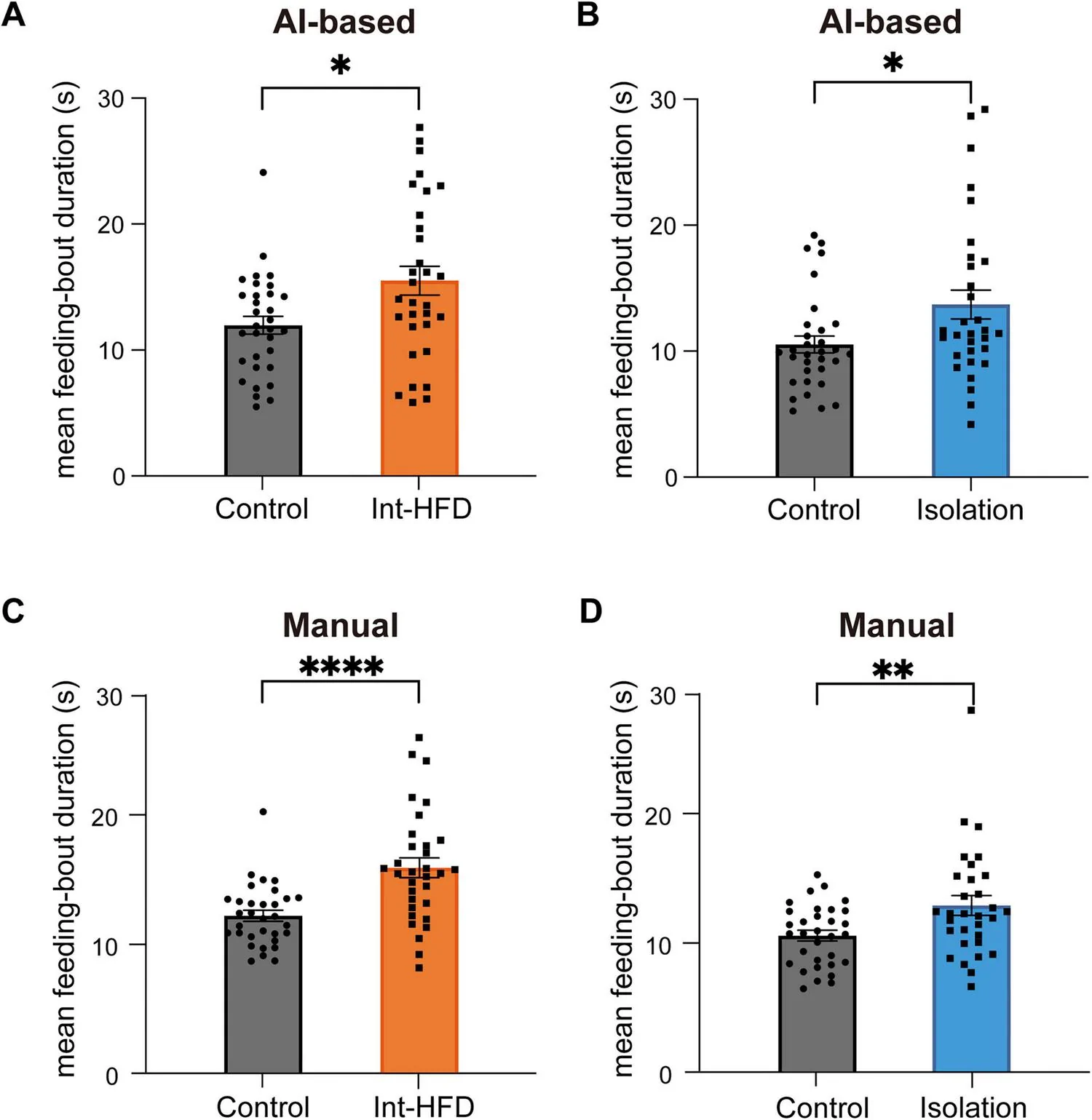
Social isolation and intermittent HFD induce protracted feeding. Mean feeding-bout duration was quantified using AI-based behavioral analysis and manual scoring. Sex composition was balanced or nearly balanced across groups. For the AI-based analysis, the intermittent HFD cohort comprised 16 male and 16 female mice in the Control group and 15 male and 16 female mice in the Int-HFD group. The social isolation cohort comprised 16 male and 16 female mice in the Control group and 16 male and 15 female mice in the Isolation group. Sex-stratified analyses are shown in Supplementary Figure 2. **(A)** AI-based analysis showed that intermittent HFD significantly increased the mean feeding-bout duration compared with controls (*n* = 32 for Control, *n* = 31 for Int-HFD, Welch’s *t*-test, *p* = 0.0110). **(B)** AI-based analysis showed that social isolation also increased mean feeding-bout duration compared with controls (*n* = 32 for Control, *n* = 31 for Isolation, Welch’s *t*-test, *p* = 0.0213). **(C)** Manual scoring confirmed that intermittent HFD increased mean feeding-bout duration compared with controls (*n* = 32 for each, Welch’s *t*-test, *p* < 0.0001). **(D)** Manual scoring confirmed that social isolation increased mean feeding-bout duration compared with controls (*n* = 32 for Control, *n* = 31 for Isolation, Welch’s *t*-test, *p* = 0.0096). Protracted feeding was defined as an increase in mean feeding-bout duration after merging classifier interruptions shorter than 7 s. *p < 0.05, **p < 0.01, ****p < 0.0001. Data are mean ± SEM. Total food intake for these sessions was reported previously (Fujioka et al., 2024; Figures 3A, B) and was not re-measured here.

**FIGURE 3:**
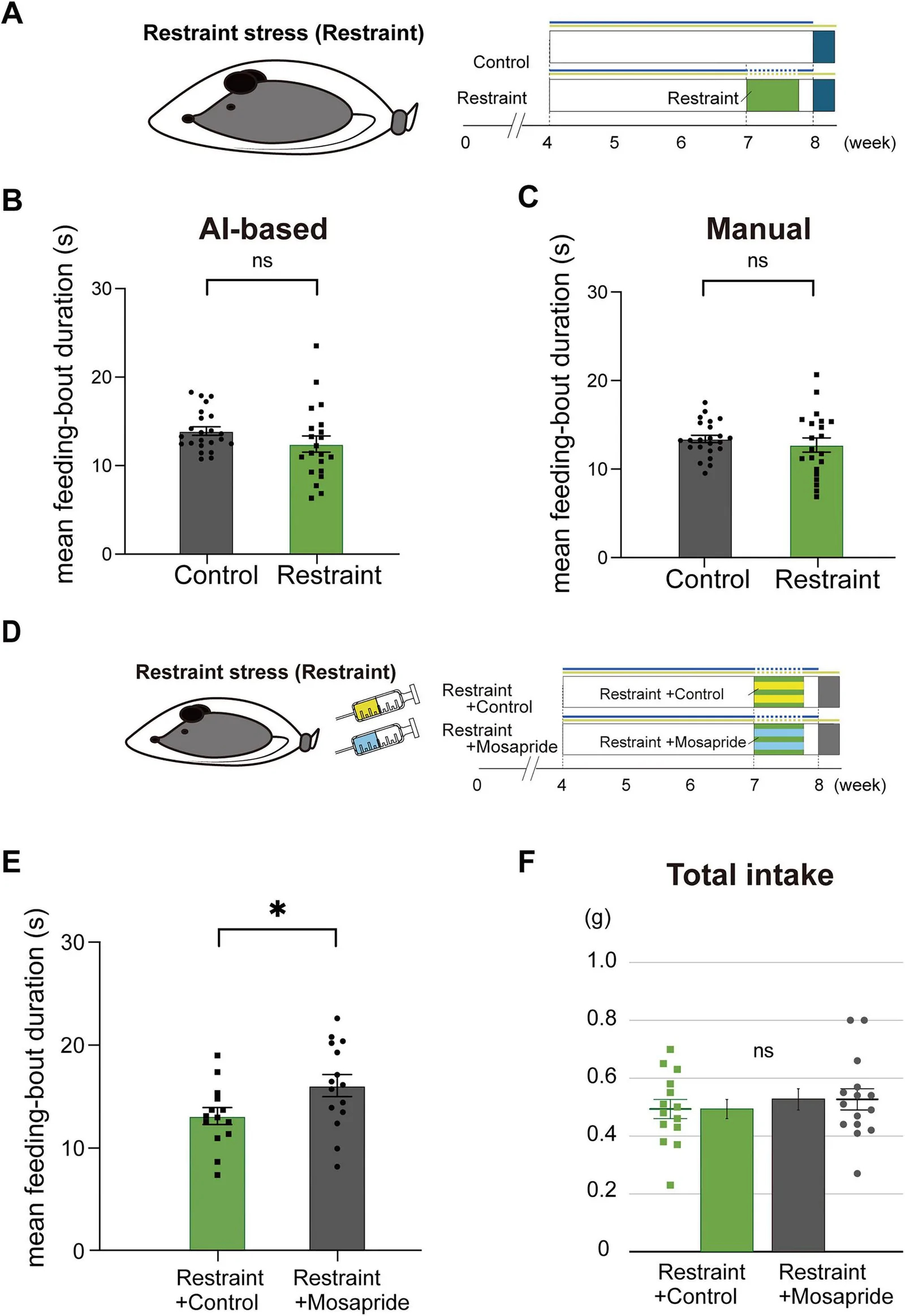
Physical restraint stress alone does not induce protracted feeding, whereas mosapride treatment increases mean feeding-bout duration in restrained mice without altering spatial fixated feeding or total intake. **(A)** Schematic illustration of the physical restraint stress paradigm. Mice in the restraint group were subjected to restraint stress during the indicated experimental period, whereas control mice were maintained under standard conditions. In the timelines, the lines and the dark box are as in Figure 1; dotted segments mark the 2-h daily restraint periods, when food and water were unavailable. **(B)** Mean feeding-bout duration was quantified using AI-based analysis in the combined male and female control and restraint cohorts. Physical restraint stress alone did not significantly alter mean feeding-bout duration compared with controls (*n* = 23 for Control, *n* = 21 for Restraint, Welch’s *t*-test, *p* = 0.1661). **(C)** Manual scoring independently confirmed that physical restraint stress did not significantly affect mean feeding-bout duration (*n* = 23 for Control, *n* = 21 for Restraint, Welch’s *t*-test, *p* = 0.4492). Each point represents one mouse. Bars indicate mean ± SEM. ns, not significant. **(D)** Schematic for experiments using mosapride with mice under restraint stress. Seven-week-old mice were subjected to 2 h of complete immobilization for five consecutive days. Mosapride citrate solution or control solvent was orally administered after each restraint period. The restraint plus mosapride cohort comprised 14 male mice in the restraint plus control group and 15 male mice in the restraint plus mosapride group. No female mice were included in this cohort. **(E)** AI-based analysis using DeepLabCut and SimBA showed that mean feeding-bout duration was significantly increased in the restraint + mosapride group compared with the restraint + control group (*n* = 14 for Restraint + Control, *n* = 15 for Restraint + Mosapride, Welch’s *t*-test, *p* = 0.0374). **(F)** Total intake during the behavioral recording period was not significantly affected by mosapride (*n* = 14 for Restraint + Control, *n* = 15 for Restraint + Mosapride, Welch’s *t*-test, *p* = 0.501). Each dot represents one mouse. Bars indicate mean ± SEM. *p < 0.05; ns, not significant. Total food intake for the sessions in panels **(B,C)** was reported previously (Fujioka et al., 2024; Figures 3A, B); the intake in panel **(F)** was measured in the present study.

**FIGURE 4:**
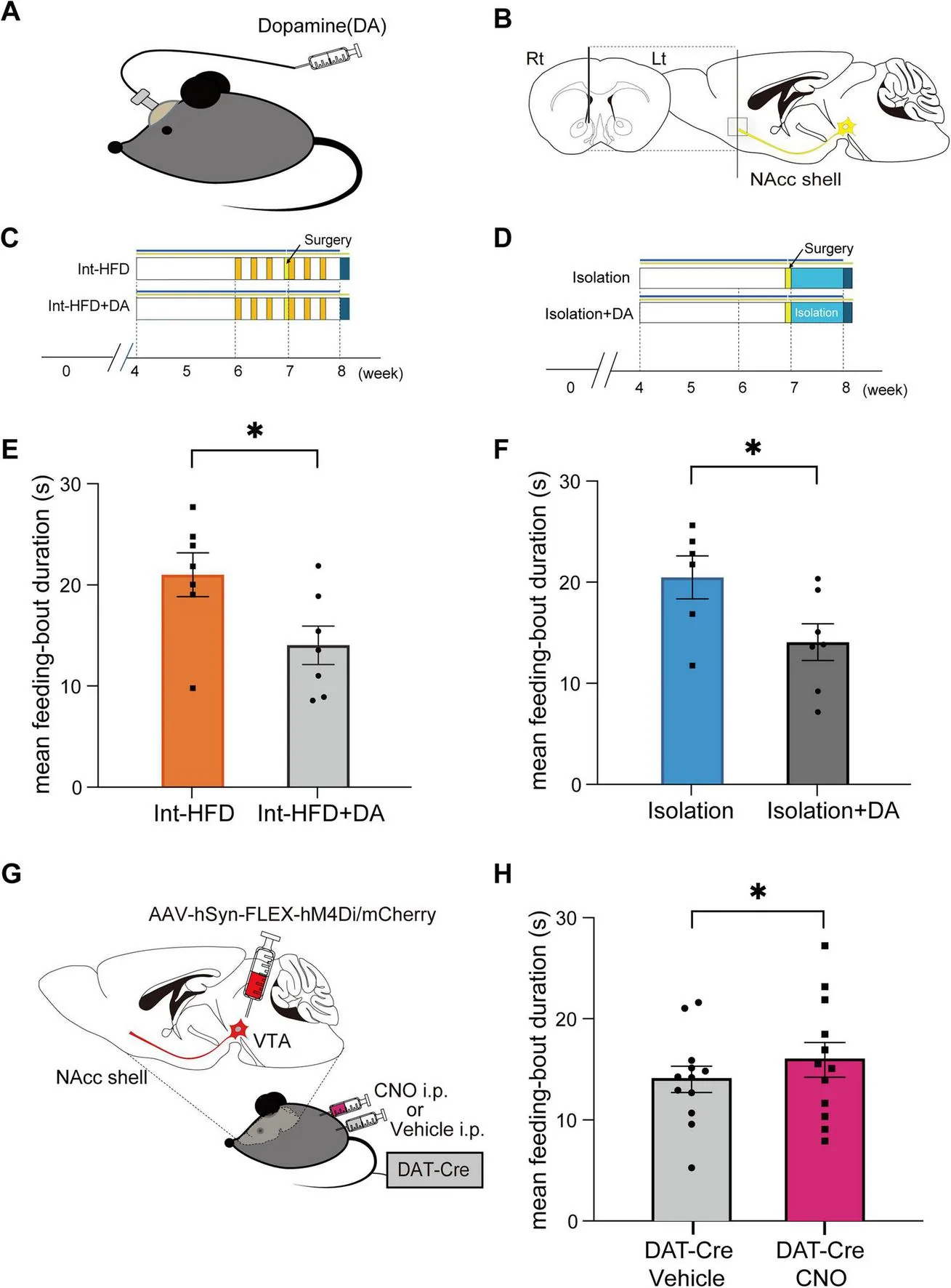
NAcc shell dopamine signaling mitigates diet- and isolation-induced protracted feeding, and inhibition of VTA dopaminergic neurons reproduces protracted feeding. **(A)** Schematic of dopamine microinfusion. **(B)** Schematic representation of the NAcc shell injection site and the mesolimbic pathway from the VTA to the NAcc shell. **(C)** Timeline of the intermittent HFD paradigm. Mice were exposed to intermittent HFD between weeks 6 and 8; surgery is marked at week 7, followed by dopamine administration. **(D)** Timeline of the social isolation paradigm. Mice underwent surgical implantation followed by isolation during weeks 7 and 8, with dopamine administration during the isolation phase. In the timelines in panels **(C,D)**, the lines and the dark box are as in Figure 1. **(E)** Among intermittently HFD-exposed mice, intra-NAcc shell dopamine infusion reduced mean feeding-bout duration compared with vehicle infusion (*n* = 7 per group, Welch’s *t*-test, *p* = 0.0332). **(F)** Among socially isolated mice, intra-NAcc shell dopamine infusion reduced mean feeding-bout duration compared with vehicle infusion (*n* = 6 for Isolation and *n* = 7 for Isolation + DA, Welch’s *t*-test, *p* = 0.0440). *p < 0.05. Data are mean ± SEM. **(G)** Schematic illustration of DREADD-mediated inhibition of VTA dopaminergic neurons. AAV-hSyn-FLEX-hM4Di/mCherry was injected into the VTA of DAT-Cre mice, and CNO or vehicle was administered intraperitoneally. **(H)** DREADD-mediated inhibition of VTA dopaminergic neurons significantly increased mean feeding-bout duration under CNO treatment compared with vehicle treatment (*n* = 12 mice, each tested under both vehicle and CNO conditions; paired *t*-test, *p* = 0.0360). Each dot represents one mouse. Bars indicate mean ± SEM. *p < 0.05. Cohorts in panels **(E,F,H)** comprised male mice only: the HFD rescue cohort comprised seven male mice per group. The isolation rescue cohort comprised six male mice in the isolation group and seven male mice in the isolation plus DA group. The DAT-Cre cohort comprised 12 male mice assessed under both vehicle and CNO conditions. No female mice were included in these cohorts. Total food intake for the sessions in panels **(E,F)** was reported previously and did not differ significantly between vehicle- and dopamine-treated mice (Fujioka et al., 2024; Supplementary Figures 3A, B).

All animal experiments were performed in accordance with the National Institutes of Health Guide for the Care and Use of Laboratory Animals and were approved by the Nagoya University Animal Experiment Committee and the Shiga University of Medical Science Animal Care and Use Committee. All methods were carried out in accordance with relevant guidelines and regulations and are reported in accordance with the ARRIVE guidelines.

### 2.2 Behavioral video recordings and AI-based temporal analysis

Feeding behavior was assessed in an arena containing four bait containers with a standard chow diet affixed to the surface, as described previously for the assessment of fixated feeding (Fujioka et al., 2024). The present study focused on the temporal organization of feeding behavior by applying a new DeepLabCut/SimBA-based analytical workflow to behavioral videos obtained in this four-bait-container assay. Across the stress, pharmacological, and DREADD cohorts, the same temporal analysis framework and bout-definition criteria were applied to the corresponding behavioral recordings.

Behavioral recordings followed the protocol established for this assay previously (Fujioka et al., 2024). Immediately before each recording, all mice, including controls, were deprived of food for 4 h, with water available during this period. Feeding behavior was then recorded for 4 h in the four-bait-container arena; water was not available during the recording session. The same procedure was applied to every group and every cohort. This standardizes pre-session food access but does not by itself ensure an equivalent state of hunger or satiety at session onset (see Discussion). During the assay, mice were allowed to move freely, and feeding sessions were recorded for 4 h using a video camera at 30 frames per second with 720p resolution. To validate the AI-based analysis, manual and AI-based measurements were obtained from the same behavioral recordings. The validation dataset comprised the 4-h recording sessions of 170 mice. Each session was stored as a variable number of sequential video files during acquisition, and all files belonging to a session were analyzed together, so validation was performed at the level of the recording session and the mouse rather than by a fixed video-file count. For each mouse, the manually determined mean feeding-bout duration was compared with the corresponding mean feeding-bout duration obtained using the AI-based analysis. Manual scoring was performed by an observer blinded to group allocation. The detailed manual scoring methods were described elsewhere (Fujioka et al., 2024).

### 2.3 Feeding behavior detection using DeepLabCut and SimBA

Automated pose estimation was performed using a ResNet-50 DeepLabCut network with shuffle 1 and snapshot 070. The tracked body parts were the left ear, right ear, nose, and tail base (DeepLabCut labels: Ear_left, Ear_right, Nose, and Tail_base). The x- and y-coordinate trajectories were median-filtered using a five-frame window, whereas the corresponding likelihood values were left unchanged. The filtered coordinate data were subsequently processed using SimBA for behavioral classification.

Frame-level feeding predictions were generated using a random-forest classifier comprising 500 trees and 165 input features. A single classifier trained using data from the intermittent HFD cohort was applied to all experimental cohorts. The final classifier was identified by the SHA-256 hash 327d41d8d86846de388b0232bc0d53960d4d44834ea6180f714936a 42b9601dc. The classifier outputs were used to identify feeding bouts, which were subsequently processed using the 7-s gap-merging procedure described in section “2.4 Definition of protracted feeding.”

The classifier-training data were obtained from one male mouse in the intermittent HFD group and consisted of 8,992 manually annotated frames, including 5,166 feeding-positive frames across six feeding events. On the held-out test set, the classifier achieved a precision of 0.994, a recall of 0.993, and an F1 score of 0.994. Because classifier predictions for the training animal were not fully out-of-sample, this animal was excluded from the manual-versus-AI regression shown in Figure 1D and from the AI-based HFD group comparison shown in Figure 2A. However, the animal was retained in the manual-only HFD comparison shown in Figure 2C because that analysis did not use classifier-derived measurements. None of the animals in the other experimental cohorts were used for classifier training.

For the manual-versus-AI comparison, the same behavioral recordings from the remaining 170 mice in the intermittent HFD, social isolation, and physical restraint experiments were evaluated manually. For each mouse, the manually determined mean feeding-bout duration was compared with the corresponding AI-derived mean feeding-bout duration. The regression therefore used one paired manual and AI-derived value per mouse.

### 2.4 Definition of protracted feeding

Using the AI-based behavioral analysis system, we defined an aberrant temporal feeding pattern termed protracted feeding. Whereas fixated feeding is characterized by an abnormal deviation in spatial bait selection, protracted feeding is characterized by an increase in mean feeding-bout duration. An initial feeding bout was defined as a continuous period classified as eating by the system. Video files belonging to the same mouse were first placed on a continuous recording-session timeline. Adjacent bouts separated by gaps shorter than 7 s were then merged and treated as a single feeding bout, including across consecutive video-file boundaries. This rule bridges brief interruptions in classifier output that do not reflect true cessation of feeding. The 7-s value functions solely as a gap-merging threshold and was not used to exclude longer inter-bout intervals from analysis. It was selected empirically by inspecting the behavioral recordings: a 5-s threshold tended to split apparently continuous feeding episodes into separate bouts, whereas a 10-s threshold tended to merge episodes that appeared behaviorally distinct, so 7 s was adopted as a practical compromise between excessive splitting and excessive merging. The mean feeding-bout duration was then calculated for each mouse. For all temporal analyses, the same 7-s gap-merging rule and per-mouse endpoint definition were applied. The principal endpoint was the per-mouse mean feeding-bout duration, which summarizes temporal feeding organization while limiting bout-level pseudoreplication. This per-mouse summary follows prior meal-pattern analyses that treat bout duration, bout number, and inter-bout intervals as informative descriptors of ingestive behavior (Zorrilla et al., 2005). In addition to the mean feeding-bout duration, four secondary parameters were derived from the same classifier output for each mouse: the mean inter-bout interval, defined as the mean duration of the intervals separating consecutive feeding bouts after gap merging; the number of feeding bouts recorded during the 4-h session; the latency to feed, defined as the interval from the start of the session to the onset of the first feeding bout; and the total feeding time, defined as the summed duration of all merged feeding bouts in the session, including the brief gaps bridged by the merging rule. These parameters are reported in the Supplementary material.

### 2.5 Stereotaxic surgery and pharmacological manipulation

To examine the contribution of NAcc shell dopamine signaling, stereotaxic surgery was performed to implant a dopamine (DA) injection port. DA was administered locally into the NAcc shell (coordinates: ML: +0.42, AP: +1.34, DV: −4.25 mm relative to bregma) to test whether dopamine supplementation reverses stress-induced prolonged feeding. Mice were anesthetized via intraperitoneal injection with a mixed anesthetic agent consisting of medetomidine (0.3 mg/kg), midazolam (4 mg/kg), and butorphanol (5 mg/kg). The dopamine solution was prepared as 3-hydroxytyramine hydrochloride: C_8_H_11_NO_2_-HCl (Tokyo Chemical Industry, Tokyo, Japan) diluted in solution buffer [0.5% ascorbic acid (ASA; Iwaki Seiyaku, Tokyo, Japan)/Ringer’s solution]. Dopamine administration was performed 5 min before behavioral analysis by injecting 0.5 μL of dopamine solution (200 μg/μL) into the NAcc shell at a flow rate of 0.25 μL/min. The injection cannula was maintained in place post-injection for 3 min. For vehicle-treated mice, the same volume of 0.5% ASA/Ringer’s solution was injected into the NAcc shell. In addition, VTA dopaminergic inhibition was examined in DAT-Cre mice using the DREADD-based strategy described previously (Fujioka et al., 2024) to test whether suppression of VTA dopaminergic activity affects the temporal organization of feeding.

For the DREADD-mediated inhibition cohort, AAV-hSyn-FLEX-hM4Di/mCherry was stereotaxically injected into the VTA of DAT-Cre mice as described previously (Fujioka et al., 2024). This construct was selected because the FLEX (double-floxed inverted open reading frame) cassette renders expression Cre-dependent and therefore confines the inhibitory DREADD hM4Di to DAT-expressing VTA neurons, hM4Di reduces neuronal activity upon CNO administration (Armbruster et al., 2007; Roth, 2016), and the mCherry reporter permits histological verification. The same construct and the same cohort were used in our previous study, in which CNO administration reduced dopamine release in the NAcc shell and reproduced fixated feeding (Fujioka et al., 2024), so the present temporal analysis is directly comparable with those published findings. We injected 0.5 μL per side of AAV-hSyn-FLEX-hM4Di/mCherry (2.0 × 10^13^ copies/mL) into the bilateral VTA regions (ML: ±0.4, AP: −3.28, DV: −4.50 mm relative to bregma) of 6-week-old DAT-Cre mice at a flow rate of 0.25 μL/min. The injection cannula was maintained in place post-injection for 3 min. Mice were anesthetized as described above. CNO [1.0 μg/g of body weight, 0.1 μg/μL in 10% DMSO (Sigma-Aldrich, St. Louis, MO)-saline mixture] was intraperitoneally administered 30 min before behavioral assessment. For vehicle treatment, the same volume of 10% DMSO-saline mixture was administered intraperitoneally. Behavioral recordings from the DREADD-mediated inhibition cohort were analyzed using the same DeepLabCut/SimBA-based temporal framework applied to the other cohorts. In this cohort, hM4Di/mCherry expression in TH-positive VTA neurons was confirmed histologically. Microdialysis after behavioral testing showed that CNO attenuated the feeding-associated increase in NAcc shell dopamine release (Fujioka et al., 2024; Figures 4B, D).

### 2.6 Mosapride administration

In our previous study, physical restraint stress produced a sympathetic and metabolic signature that included elevated plasma norepinephrine and reduced body weight in both sexes and reduced food intake in female mice. We therefore tested whether the peripheral autonomic and gastrointestinal state modulates the detectability of temporal feeding abnormalities after restraint stress. Mosapride citrate solution or control solvent was orally administered after each restraint period. Mosapride citrate was suspended in 0.5% tragacanth gum at a concentration of 0.5 mg/mL, and 2 mL/kg of the suspension was given by stomach tube. Mosapride is a peripheral 5-HT4 receptor agonist that enhances gastrointestinal motility. Because stress can alter the gut-brain and autonomic pathways that regulate gastrointestinal function, this intervention was used as a pharmacological probe of peripheral constraint rather than as proof of a specific norepinephrine-mediated mechanism (Taché and Perdue, 2004; Mayer, 2011). Temporal feeding patterns, spatial fixated feeding, and total intake were assessed after treatment. The aim was to test whether supporting gastrointestinal motility modifies feeding-bout duration under restraint stress. Gastrointestinal motility and sympathetic activity were not directly measured in this experiment. The mosapride experiment was performed in male mice only, so that this cohort would be directly comparable with the male-only intra-NAcc shell infusion and chemogenetic cohorts (Fujioka et al., 2024) on which the mechanistic interpretation of this study rests.

### 2.7 Computer hardware and software

DeepLabCut and SimBA were used for pose estimation and behavioral classification, respectively. The exact DeepLabCut and SimBA release numbers used during the original analyses were not retained in the archived output files. The final replay of the serialized random-forest classifier was performed in Python 3.9.6 using scikit-learn 1.2.2. Metadata stored in the serialized classifier indicated that scikit-learn 0.22.2 was the version used when the classifier was saved. The analysis computer was equipped with an Intel i9-10900 CPU, an NVIDIA RTX 5080 GPU, and 128 GB of memory and ran Ubuntu 24.04 LTS. ChatGPT (GPT-5, OpenAI) was used for technical assistance with file management, scripting, and troubleshooting during data analysis. All analytical procedures and decisions were determined and verified by the authors.

### 2.8 Data and statistical analyses

Linear regression analysis was used to evaluate the association between the manually determined mean feeding-bout duration and the AI-derived mean feeding-bout duration. The regression was performed using one paired manual and AI-based value per mouse, pooled across the intermittent HFD, social isolation, and physical restraint experiments (*n* = 170 mice), after excluding the mouse used for classifier training. Differences between groups, including mean feeding-bout duration, preference ratio, and total intake, were analyzed using Welch’s *t*-test, without assuming equal variances. The chemogenetic experiment shown in Figure 4H was the only within-subject comparison: each hM4Di-expressing DAT-Cre mouse received both the vehicle and CNO, and the two conditions were recorded in the same animals on separate days in a counterbalanced order. The two measurements obtained from each mouse were therefore compared using a paired *t*-test, and *n* denotes the number of mice rather than the number of recordings. All tests were two-tailed. The test applied to each comparison is indicated in the corresponding figure legend. Statistical significance was set at *p* < 0.05. Data are expressed as mean ± SEM.

For statistical comparisons of mean feeding-bout duration, each mouse was treated as the experimental unit, and the per-mouse mean feeding-bout duration was used for analysis. Individual feeding bouts or video frames were not treated as independent biological replicates. Exact sample sizes and statistical tests are indicated in the figure legends.

## 3 Results

### 3.1 AI-based analysis detects temporal feeding behavior patterns

To investigate how feeding behavior patterns are temporally organized, we used the same stress paradigms examined in our previous study: social isolation, a major inducer of anxiety; intermittent high-fat diet (HFD), which provokes binge-like eating behaviors; and physical restraint, which mimics the physical stress caused by confinement (Figures 1A, B, 3A). We retained these models because, in our previous work, they produced divergent quantitative feeding outcomes, including increased, unchanged, and reduced intake, while sharing spatial fixated feeding. This allowed us to test whether the temporal organization of feeding also shows a convergent stress-related alteration.

Feeding sessions were recorded at 30 frames per second for 4 h in the same four-bait-container arena. DeepLabCut was used to track body parts, and SimBA was used to classify eating behavior from the pose-estimation data (Figure 1C). To validate the AI-based system, the AI-derived mean feeding-bout duration for each mouse was compared with the corresponding manually determined mean feeding-bout duration obtained from the same recordings. Linear regression analysis of the paired per-mouse values, pooled across the intermittent HFD, social isolation, and physical restraint experiments, showed a significant positive association between the two methods (R^2^ = 0.5819, *p* < 0.0001, *n* = 170; Figure 1D). The automated system therefore reproduced the manual estimates of bout duration at the group level and allowed high-throughput quantification of temporal feeding structure. The residual scatter indicates that the two methods are not interchangeable for individual animals.

### 3.2 Social isolation and intermittent HFD induce protracted feeding

Using the AI-based analysis, we first quantified temporal feeding behavior patterns in mice exposed to the stressors. In control mice, feeding was organized into short, discrete bouts. In contrast, mice subjected to intermittent HFD or social isolation showed significantly prolonged feeding-bout durations compared with controls (intermittent HFD, *p* = 0.0110; social isolation, *p* = 0.0213; Welch’s *t*-test; Figures 2A, B). We defined this stress-associated prolongation of individual feeding bouts as protracted feeding. This phenotype is a temporal deviation in feeding behavior patterns, complementary to the spatial deviation seen in fixated feeding. In our previous study, in which the same recording sessions were used to quantify food consumption, intermittent HFD significantly increased total intake in male mice, with a similar trend in females, whereas social isolation produced no consistent change in intake (Fujioka et al., 2024; Figures 3A, B). Both stressors nevertheless prolonged individual feeding bouts in the present analysis, so the temporal feeding abnormality is unlikely to be simply a surrogate for increased consumption. Manual scoring independently confirmed the prolonged feeding duration observed in the intermittent HFD and social isolation mice (*p* < 0.0001 and *p* = 0.0096, respectively; Welch’s *t*-test; Figures 2C, D).

Re-analysis of the same recordings showed that the prolongation of feeding bouts was not accompanied by statistically significant changes in the number of bouts, the latency to the first bout, or the mean inter-bout interval in either model (Supplementary Figures 1A, B). Total feeding time was greater in socially isolated mice (*p* = 0.0269) and was numerically greater in the intermittent HFD model without reaching statistical significance (*p* = 0.1963), consistent with longer bouts without a detectable change in bout number. Because all animals were food-deprived for 4 h immediately before the session (section “2.2 Behavioral video recordings and AI-based temporal analysis”), the unchanged latency indicates that the propensity to begin feeding was not detectably altered. The temporal phenotype therefore appears to reside in the maintenance of initiated feeding episodes rather than in the initiation of feeding or the spacing of episodes, although these secondary parameters are unadjusted descriptive comparisons and the per-mouse mean feeding-bout duration remains the primary endpoint.

Sex-stratified analyses showed significantly prolonged feeding-bout duration in female but not male mice exposed to intermittent HFD (males, *p* = 0.0720; Supplementary Figure 2A), and in both male and female mice exposed to social isolation. After physical restraint alone, the difference did not reach statistical significance in either sex (Supplementary Figure 2). The effect was in the same direction in males and females in both models, but these sex-stratified analyses are underpowered by design, because the experiments were powered for the pooled comparisons; we therefore treat the concordant direction as suggestive rather than as evidence that the phenotype is equivalent in the two sexes.

### 3.3 Physical restraint stress alone does not significantly alter mean feeding-bout duration, whereas mosapride increases it in restrained mice

We next examined whether physical restraint stress alone was sufficient to induce protracted feeding. Male and female restraint cohorts were combined for the AI-based analysis using DeepLabCut and SimBA (Figure 3A). In contrast to intermittent HFD exposure and social isolation, physical restraint stress alone did not increase the mean feeding-bout duration compared with controls. Mean feeding-bout duration was slightly lower in restrained mice than in controls, and the difference was not statistically significant (Control, *n* = 23; Restraint, *n* = 21; Welch’s *t*-test, *p* = 0.1661; Figure 3B). Manual scoring likewise showed no significant difference in mean feeding-bout duration between control and restraint mice (Figure 3C). Total feeding time was lower in restrained mice (*p* = 0.0353), whereas bout number, latency to the first bout and mean inter-bout interval did not differ significantly (Supplementary Figure 1C). This unadjusted secondary comparison is descriptive and does not alter the finding that restraint alone did not prolong mean feeding-bout duration. Physical restraint stress alone therefore does not robustly induce protracted feeding under the present conditions. In the same recording sessions analyzed previously, physical restraint reduced total intake significantly in female mice, with a similar tendency in males (Fujioka et al., 2024; Figures 3A, B). Restraint therefore reduced the amount consumed without prolonging mean feeding-bout duration under the present conditions. One possibility is that the peripheral physiological responses to restraint stress constrain stress-related temporal feeding abnormalities, or reduce their detectability.

In our previous study, the restraint group showed metabolic and physiological changes associated with sympathetic nervous system activation, including increased plasma norepinephrine, increased blood glucose, increased body temperature, decreased spleen weight, and increased brown adipose tissue (Fujioka et al., 2024). We therefore hypothesized that restraint stress engages stress-related feeding-pattern mechanisms and at the same time imposes peripheral autonomic and gastrointestinal constraints. Such constraints would limit the maintenance of feeding episodes and reduce the detectability of protracted feeding. This interpretation is consistent with evidence that stress influences both mesolimbic dopamine function and the gut-brain and autonomic regulation of ingestive physiology (Taché and Perdue, 2004; Mayer, 2011).

To test this possibility, mice in the restraint stress group were treated with mosapride, a peripheral 5-HT4 receptor agonist that enhances gastrointestinal motility (Figure 3D). AI-based analysis using DeepLabCut and SimBA showed a significant increase in mean feeding-bout duration in the Restraint + Mosapride group compared with the Restraint + Control group (Welch’s *t*-test, *p* = 0.0374; Figure 3E). In this cohort, mosapride also increased total feeding time (*p* = 0.0384), without significant changes in bout number, latency, or inter-bout interval (Supplementary Figure 1D). Mosapride did not significantly alter spatial fixated feeding (Supplementary Figure 3) or total intake during the behavioral recording period (Figure 3F). Mosapride therefore prolonged feeding bouts in restrained mice. Because no non-stressed mosapride-treated group was included, these data do not establish whether the effect is specific to restraint stress.

### 3.4 Mesolimbic dopamine signaling bidirectionally regulates protracted feeding

Our previous study linked stress-impaired dopamine responses in the NAcc shell to fixated feeding (Fujioka et al., 2024). We therefore asked whether NAcc shell dopamine signaling also regulates temporal feeding behavior patterns. Among intermittently HFD-exposed mice, intra-NAcc shell dopamine administration significantly reduced mean feeding-bout duration (Welch’s *t*-test, *p* = 0.0332; Figures 4A–C, E). Among socially isolated mice, intra-NAcc shell dopamine administration also significantly reduced mean feeding-bout duration (Welch’s *t*-test, *p* = 0.0440; Figures 4A, B, D, F). Total intake during these recording sessions was reported previously for the same cohorts and did not differ significantly between vehicle- and dopamine-treated mice in either stress model (Fujioka et al., 2024; Supplementary Figures 3A, B: intermittent HFD, *p* = 0.486; social isolation, *p* = 0.133). The reduction in bout duration after dopamine infusion is therefore unlikely to be secondary to reduced consumption.

These results indicate that NAcc shell dopamine supplementation reduces protracted feeding among intermittently HFD-exposed and socially isolated mice. The temporal feeding abnormalities detected here are therefore unlikely to be explained solely by altered food access or non-specific behavioral slowing. They are instead consistent with regulation by NAcc shell dopamine signaling, in line with the reward-pathway framework described for fixated feeding.

Finally, we asked whether inhibition of VTA dopaminergic neurons affected the temporal organization of feeding. AAV-hSyn-FLEX-hM4Di/mCherry was injected into the VTA of DAT-Cre mice, and CNO was administered systemically to inhibit VTA dopaminergic neuronal activity (Figure 4G). AI-based quantification showed that VTA dopaminergic inhibition increased mean feeding-bout duration compared with vehicle treatment (paired *t*-test, *p* = 0.0360; Figure 4H). In this cohort, CNO treatment also significantly reduced total food intake compared with vehicle (paired *t*-test, *p* = 0.0075; Supplementary Figure 4), so the prolongation of feeding bouts occurred despite a reduction, rather than an increase, in the amount consumed. Reduced VTA dopaminergic activity can therefore prolong feeding bouts. This is consistent with prior work linking VTA and mesoaccumbens dopamine signaling to stress responsiveness, reward motivation, and consummatory behavior (Salamone and Correa, 2012; Holly and Miczek, 2016). Together with the NAcc shell dopamine rescue experiments, these findings indicate that mesolimbic dopamine signaling contributes to the temporal organization of feeding behavior as well as to the spatial organization reflected in fixated feeding. No significant changes in bout number, latency or mean inter-bout interval were detected in the intra-NAcc shell dopamine or DREADD cohorts. Total feeding time was lower after dopamine infusion in the isolation cohort (*p* = 0.0338), but did not differ significantly in the HFD rescue or DAT-Cre cohorts (Supplementary Figures 5A–C). These unadjusted secondary comparisons are descriptive.

## 4 Discussion

Eating behavior is regulated by brain circuits that overlap with the reward system, where homeostatic, hedonic, and motivational signals interact to shape appetite (Saper et al., 2002; Lutter and Nestler, 2009; Berridge et al., 2010; Rossi and Stuber, 2018). Whereas quantitative intake is governed largely by hypothalamic and peripheral metabolic signals, qualitative feeding patterns are a meaningful endpoint in their own right (Zorrilla et al., 2005), yet their underlying biology remains incompletely understood.

Using an automated system based on DeepLabCut and SimBA, we found that social isolation and intermittent high-fat diet (HFD) exposure induced deviations in temporal feeding patterns, which we define as “protracted feeding.” This extends our previous concept that feeding-pattern variation, rather than total intake, can index stress- and disease-associated reward-system changes (Fujioka et al., 2024). These stress models produced divergent intake outcomes: HFD tended to increase intake, isolation changed it little, and restraint tended to reduce it.

The convergence of spatial and temporal feeding-pattern abnormalities across these divergent outcomes suggests that pattern analysis can reveal reward-system dysfunction not captured by intake alone. Whereas fixated feeding reflects a spatial deviation in bait approach, protracted feeding reflects longer individual feeding bouts rather than increased consumption.

Our AI-based system quantifies protracted feeding at higher throughput than manual scoring, which is labor-intensive and open to observer bias. The association between the two methods across the full cohort was moderate (R^2^ = 0.5819, *n* = 170). Combining DeepLabCut pose estimation with SimBA supervised classification yields an interpretable workflow based on transparent body-part coordinates and user-curated events (Mathis et al., 2018; Goodwin et al., 2024), rather than an opaque end-to-end classifier, an advantage for translational applications. Whether the workflow transfers to other behaviors remains to be tested (Pereira et al., 2020).

Our data also point to an interplay between the peripheral autonomic nervous system and feeding patterns. Restraint did not initially induce protracted feeding, despite producing sympathetic activation with markedly elevated plasma norepinephrine in our previous study (Fujioka et al., 2024). We therefore hypothesized that restraint-associated autonomic and gastrointestinal states constrain sustained feeding and mask the temporal abnormality (Taché and Perdue, 2004; Mayer, 2011). The prokinetic mosapride, a peripheral 5-HT4 agonist, prolonged feeding bouts in restrained mice without altering total intake or fixated feeding. This result is consistent with a peripheral state gating the detectability of stress-related temporal abnormalities, but it does not establish a direct causal mechanism.

These findings also extend the central circuit framework of our previous study. There, stress-induced fixated feeding was linked to impaired NAcc shell dopamine responses and was reproduced by DREADD-mediated inhibition of VTA dopaminergic neurons (Salamone and Correa, 2012; Holly and Miczek, 2016; Fujioka et al., 2024). Here, NAcc shell dopamine administration attenuated protracted feeding, and reanalysis of the same DREADD videos showed that VTA inhibition also prolonged bouts, implicating the VTA dopaminergic–NAcc shell axis in the temporal organization of feeding.

Hypothalamic homeostatic and mesolimbic hedonic pathways jointly determine feeding quality (Rossi and Stuber, 2018). We found altered temporal microstructure without consistent intake changes, so protracted feeding may help dissociate hedonic from homeostatic disturbances *in vivo*.

Protracted feeding may also reflect behavioral inflexibility, a difficulty in terminating an ongoing action. Inflexibility is a cross-diagnostic dimension relevant to anxiety, obsessive-compulsive, and eating disorders (Johnson and Kenny, 2010; Robbins et al., 2012; Gillan et al., 2016). Human feeding microstructure has been linked to reward dysfunction (Sasaki, 2017), so protracted feeding may be a preclinical readout for inflexible or compulsive feeding.

Several limitations remain. First, regions beyond VTA dopaminergic activity and NAcc shell dopamine signaling may contribute to the temporal organization of feeding. Second, the present temporal analysis was based on behavioral videos originally recorded in the four-bait-container feeding assay rather than on prospectively designed independent replication cohorts. Future studies should validate protracted feeding using newly acquired recordings analyzed with the same temporal pipeline. Third, mean feeding-bout durations in the concurrently run, non-surgical comparator groups were broadly comparable across cohorts. In the AI-based analyses, comparator means were 10.5 s for social-isolation controls, 11.9 s for intermittent-HFD controls, 13.1 s for the Restraint + Control group in the mosapride experiment, and 13.8 s for physical-restraint controls. In the manual analyses, comparator means were 10.8 s for social-isolation controls, 12.4 s for intermittent-HFD controls, and 13.4 s for physical-restraint controls. Thus, the highest comparator mean in both analysis types occurred in the physical-restraint cohort, whereas the mosapride cohort was included only in the AI-based analysis. Absolute values were higher in the cohorts that underwent stereotaxic surgery and cannula implantation (Figures 4E, F). Those panels compared stressed mice with and without dopamine administration rather than including a non-stressed control group, so we cannot determine whether surgery shifted the entire cohort upward or whether the stress effect was larger in these animals. Because each pharmacological or chemogenetic manipulation was compared only with its concurrently run comparator, these differences in absolute values do not affect the within-experiment comparisons. Fourth, gastrointestinal motility and sympathetic activity were not directly measured in the mosapride experiment, so the gating mechanism remains inferential. Fifth, generalization across recording environments and strains requires further validation (Pereira et al., 2020). Sixth, all animals were food-deprived for 4 h immediately before recording, which standardizes pre-session food access; because the stress paradigms alter food intake themselves, however, this does not guarantee equivalent hunger or satiety at session onset, and a contribution of differing internal state cannot be excluded. Seventh, the mosapride experiment did not include a non-stressed mosapride-treated group, so it cannot distinguish an effect specific to restraint stress from a stress-independent effect of the drug on feeding-bout duration. Eighth, the mosapride, intra-NAcc shell dopamine and chemogenetic cohorts comprised male mice only: the latter two are re-analyses of previously acquired male cohorts (Fujioka et al., 2024), and the mosapride cohort was newly acquired as a male-only probe to remain comparable with them. The behavioral phenotype these experiments address was examined in both sexes, and the direction of the effect was consistent in males and females (Figure 2 and Supplementary Figure 2); the sex-stratified comparisons were, however, underpowered, so they do not establish equivalence between the sexes, and whether the mechanisms examined here operate similarly in female mice remains to be tested. Finally, combining this analysis with established compulsivity assays will be needed to define the overlap among these behavioral dimensions.

In summary, social isolation and intermittent HFD exposure alter the temporal structure of feeding, and mosapride treatment prolongs feeding bouts in restrained mice, although the stress specificity of that effect remains to be established. Together with fixated feeding, protracted feeding indicates that stress-impaired reward-pathway function disrupts the spatial and temporal organization of feeding. Feeding-pattern analysis may therefore provide a sensitive behavioral readout of reward-system dysfunction.

## Data Availability

The source data and custom analysis code supporting the findings of this study are publicly available in the GitHub repository (https://github.com/gxkjj/protracted-feeding-ai-analysis) and permanently archived at Zenodo (https://doi.org/10.5281/zenodo.21775568). Manually recorded data not included in the repository are available from the corresponding author on reasonable request.
