# Supplementary material for: Automated temporal analysis reveals stress-induced protracted feeding regulated by mesolimbic dopamine in mice
Source: Front Neurosci. 2026 Oct 20;20:1959447. doi: 10.3389/fnins.2026.1959447 (PMC13727574; doi:10.3389/fnins.2026.1959447)
Supplement: Supplementary file 1 [file Data_Sheet_1.pdf]

## Supplementary Figure Legends

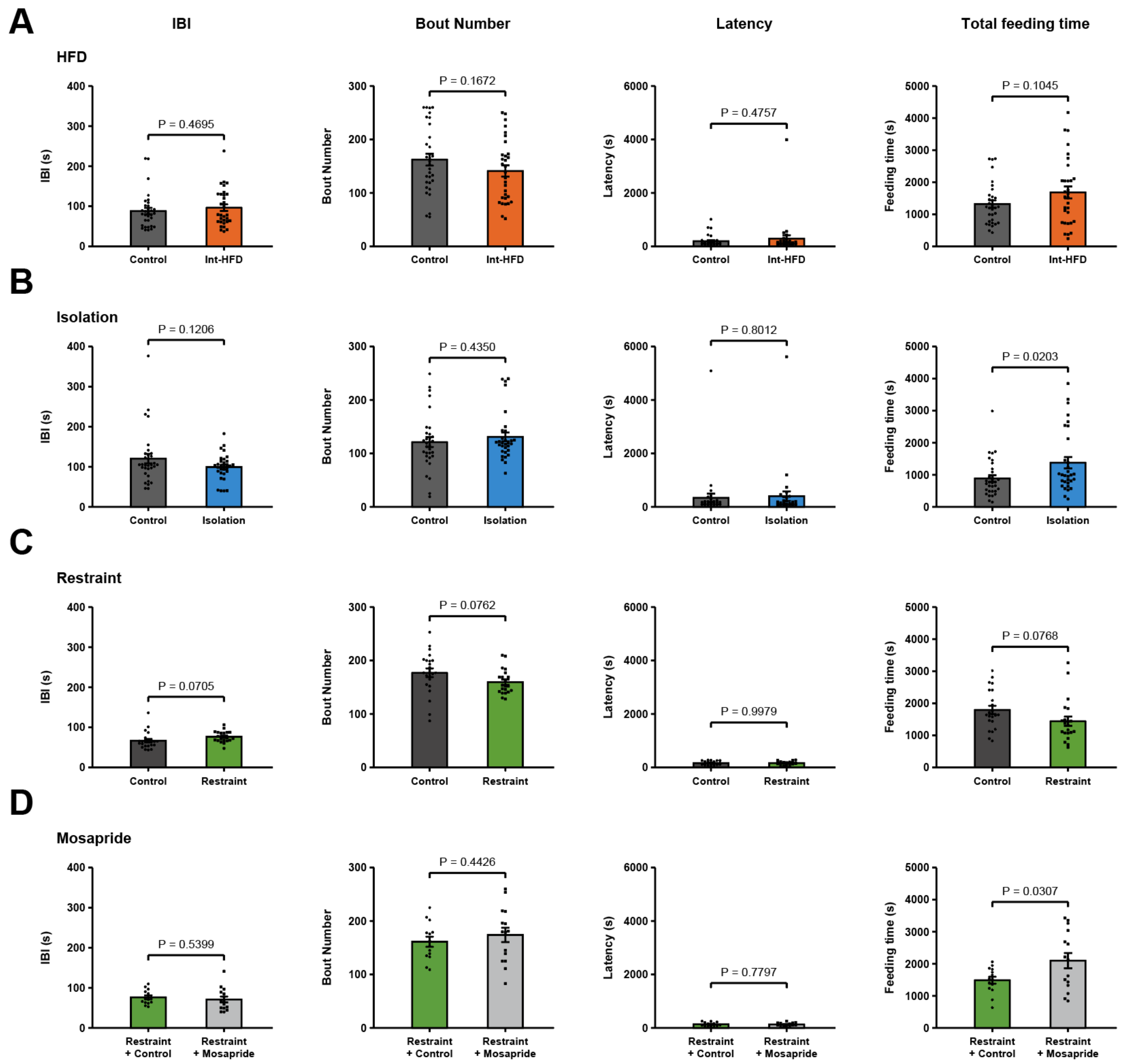


**Supplementary Figure S1 |** Additional feeding microstructure parameters across experimental conditions. Mean inter-bout interval (IBI), bout number, latency to the first feeding bout, and total feeding time are shown from left to right in each row. (A) Control and intermittent high-fat diet (Int-HFD) mice (n = 32 for Control, n = 31 for Int-HFD). (B) Control and socially isolated mice, with males and females pooled (n = 32 for Control, n = 31 for Isolation). (C) Control and restrained mice (n = 23 for Control, n = 21 for Restraint). (D) Restrained mice receiving control treatment or mosapride (n = 14 for Restraint + Control, n = 15 for Restraint + Mosapride). Each symbol represents one mouse. Bars and error bars indicate mean ± SEM. Comparisons used two-tailed Welch's t tests. Unadjusted P values are shown in the graphs; because four parameters were compared in seven cohorts across Supplementary Figures S1 and S5, individually significant secondary outcomes are treated as descriptive.

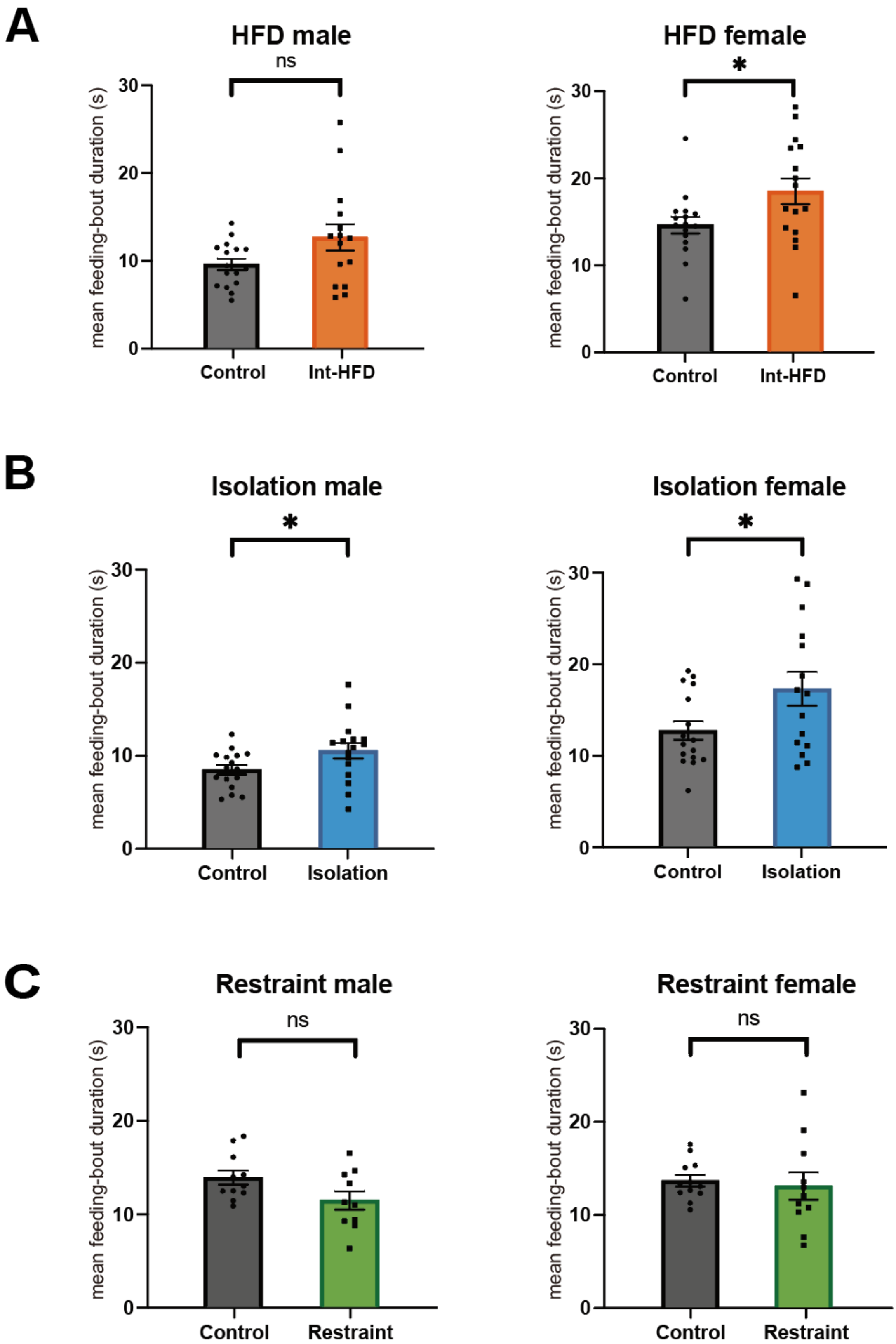


**Supplementary Figure S2 |** Sex-stratified analysis of protracted feeding across stress models. Mean feeding-bout duration was analyzed separately in male and female mice exposed to intermittent HFD, social isolation, or physical restraint stress. (A) In the

intermittent HFD model, mean feeding-bout duration was not significantly different between Control and Int-HFD male mice (Control, n = 16; Int-HFD, n = 15; Welch's t test, $p = 0.0720$), whereas it was significantly increased in Int-HFD female mice (n = 16 per group; Welch's t test, $p = 0.0380$). (B) In the social isolation model, male and female mice showed a significant increase in mean feeding-bout duration (Isolation male, n = 16 per group, Welch's t test, $p = 0.0470$; Isolation female, n = 16 for Control, n = 15 for Isolation, Welch's t test, $p = 0.0410$). (C) In the physical restraint stress model, no significant difference was detected in either sex (Restraint male, n = 11 for Control and n = 10 for Restraint, Welch's t test, $p = 0.0684$; Restraint female, n = 12 for Control and n = 11 for Restraint, Welch's t test, $p = 0.7205$). Each dot represents one mouse. Bars indicate mean ± SEM. *$p < 0.05$; ns, not significant. These sex-stratified comparisons are underpowered relative to the pooled analyses shown in Figure 2, for which the experiments were powered; the concordant direction of the effect in males and females should therefore not be taken as evidence that the phenotype is equivalent in the two sexes.

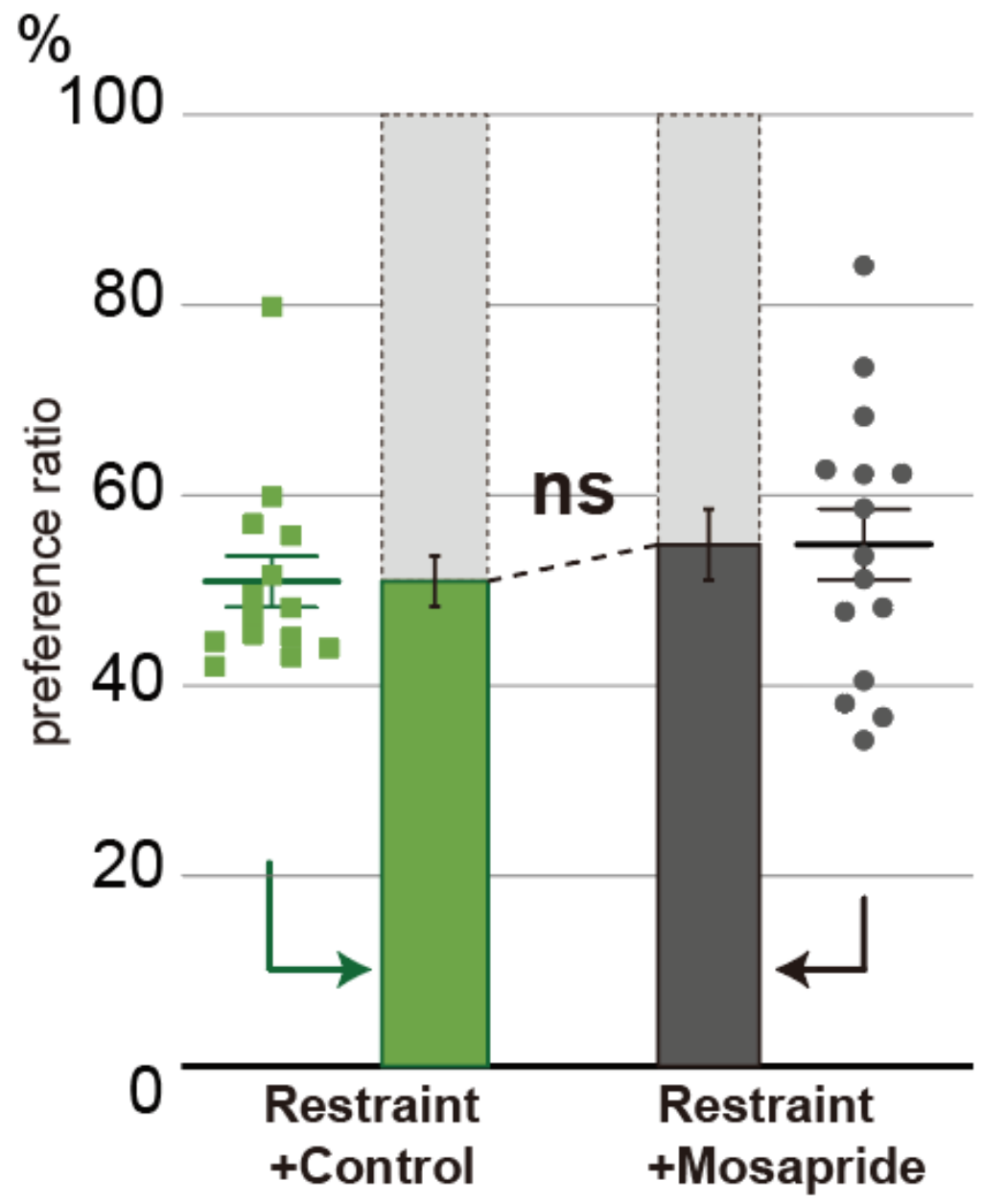


**Supplementary Figure S3 |** Spatial feeding preference after mosapride treatment. Spatial feeding patterns were assessed by quantifying the duration of time spent at each bait source in Restraint + Control and Restraint + Mosapride mice ($n = 14$ for Restraint + Control, $n = 15$ for Restraint + Mosapride, Welch's t test, $p = 0.419$). Each dot represents one mouse. Bars indicate mean ± SEM. ns, not significant.

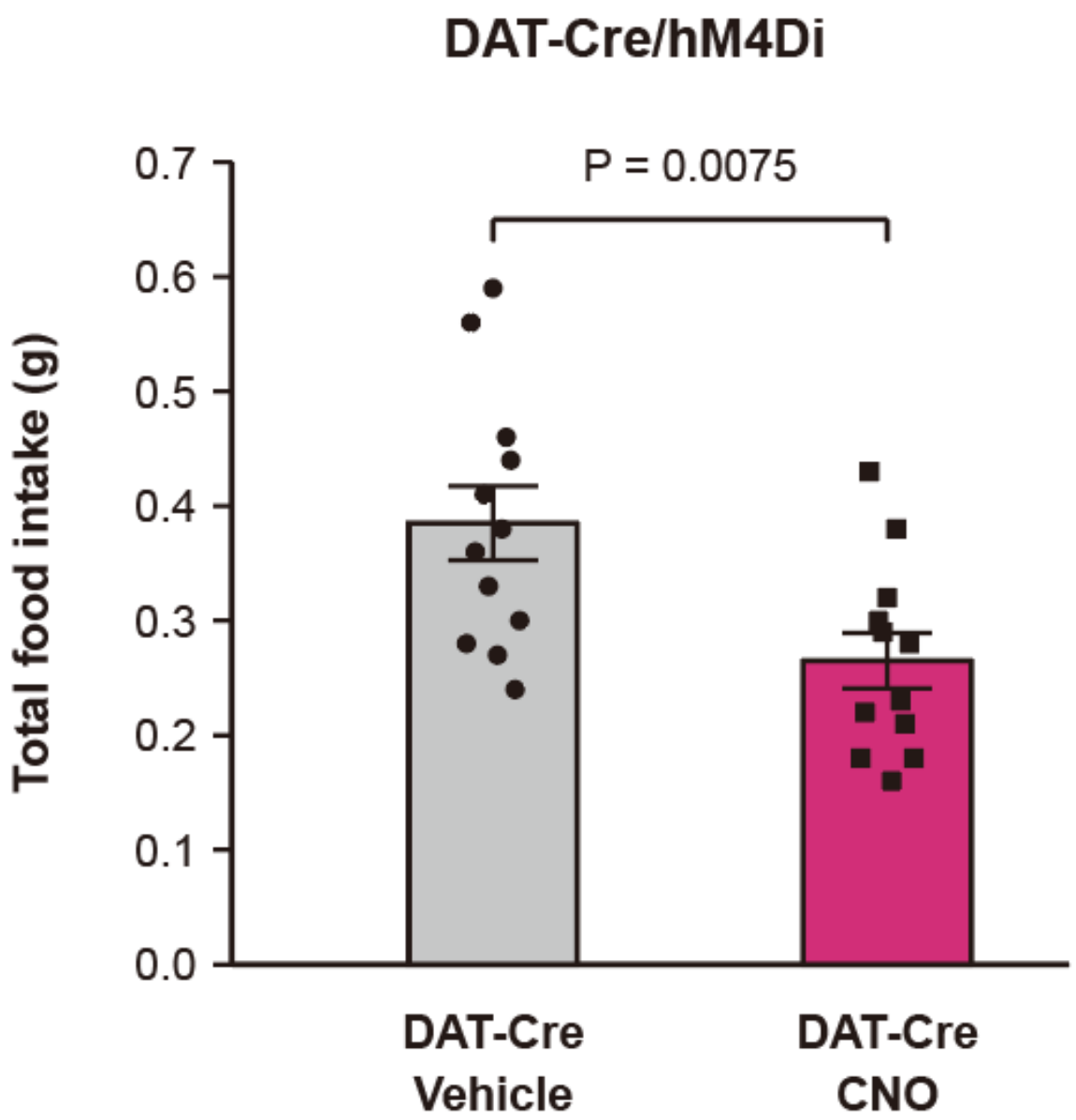


**Supplementary Figure S4 |** Total food intake during chemogenetic inhibition of VTA dopaminergic neurons. Total food intake was quantified during the 4-h recording session in DAT-Cre mice expressing hM4Di in the VTA, in the same cohort shown in Figure 4H. CNO treatment significantly reduced total food intake compared with vehicle ($n = 12$ male mice, each tested under both vehicle and CNO conditions; paired t test, $p = 0.0075$). Each dot represents one mouse. Bars indicate mean ± SEM. The exact P value is shown in the graph.

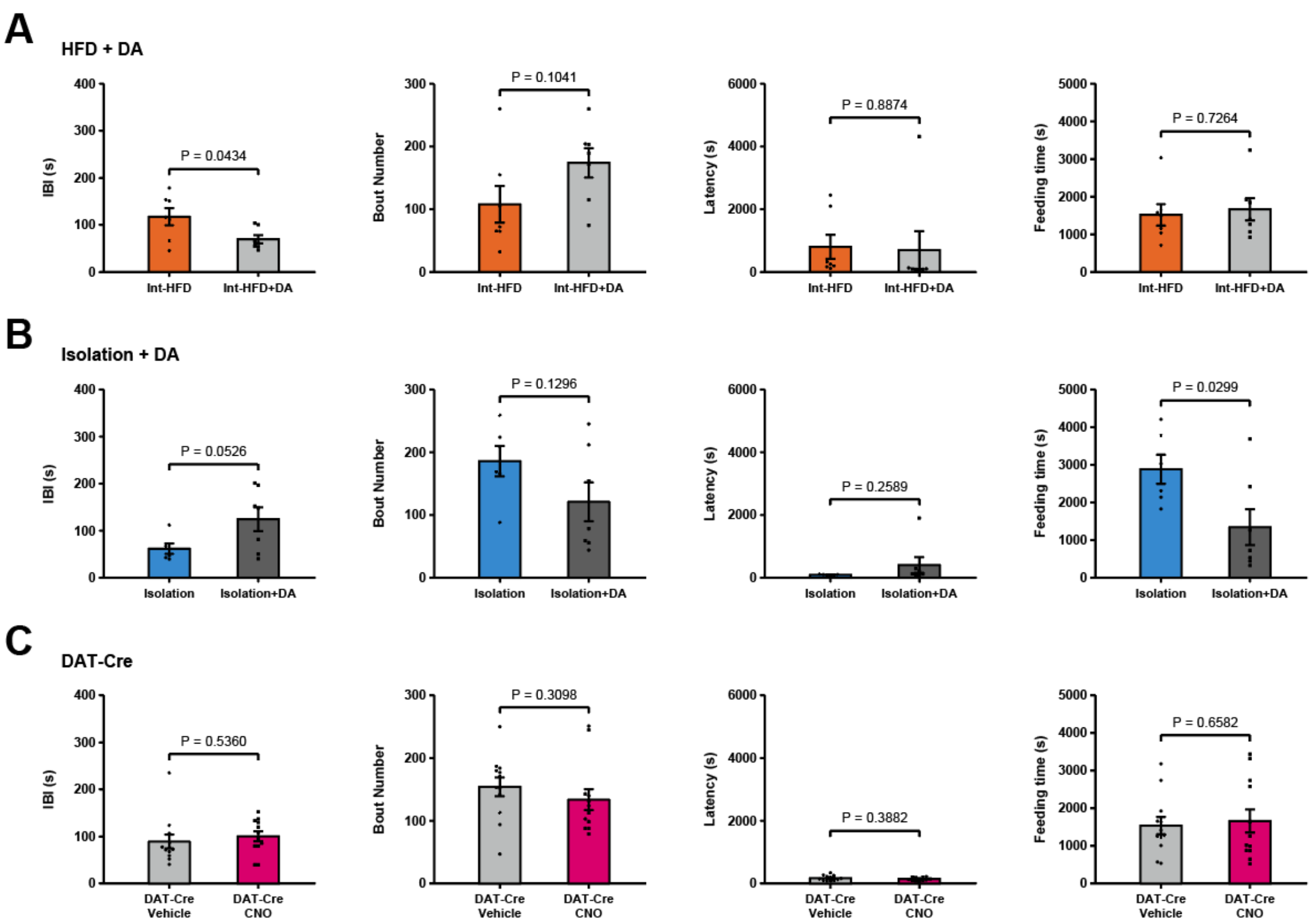


**Supplementary Figure S5 |** Additional feeding microstructure parameters in the intra-NAcc shell dopamine and chemogenetic cohorts. Mean inter-bout interval (IBI), bout number, latency to the first feeding bout, and total feeding time are shown from left to right in each row. (A,B) Dopamine (DA) infusion into the nucleus accumbens shell in Int-HFD mice (n = 7 per group) and socially isolated mice (n = 6 for Isolation, n = 7 for Isolation + DA). (C) Vehicle and clozapine N-oxide (CNO) conditions in DAT-Cre mice expressing hM4Di (n = 12 paired mice). Each symbol represents one mouse. Bars and error bars indicate mean ± SEM. Comparisons used two-tailed Welch’s t tests (A,B) or a paired t test (C). Unadjusted P values are shown in the graphs; because four parameters were compared in seven cohorts across Supplementary Figures S1 and S5, individually significant secondary outcomes are treated as descriptive.
